# Stereotactic Body radiotherapy and pedicLE screw fixatioN During one hospital visit for patients with symptomatic unstable spinal metastases: a randomized trial (BLEND RCT) using the Trials within Cohorts (TwiCs) design

**DOI:** 10.1186/s13063-023-07315-y

**Published:** 2023-05-04

**Authors:** E. H. Huele, J. M. van der Velden, N. Kasperts, W. S. C. Eppinga, J. P. C. Grutters, B. B. M. Suelmann, A. A. Weening, D. Delawi, S. C. C. M. Teunissen, H. M. Verkooijen, J. J. Verlaan, R. Gal

**Affiliations:** 1 Division of Imaging and Oncology, University Medical Center Utrecht, Utrecht University, Utrecht, the Netherlands; 2 Department of Radiation Oncology, University Medical Center Utrecht, Utrecht, the Netherlands; 3 Department for Health Evidence, Radboud University Medical Center, Nijmegen, The Netherlands; 4 Department of Medical Oncology, University Medical Center Utrecht, Utrecht, the Netherlands; 5 Department of Orthopedic Surgery, Diakonessenhuis, Utrecht, The Netherlands; 6 Department of Orthopedic Surgery, St. Antoniusziekenhuis, Nieuwegein, The Netherlands; 7 Julius Center for Health Sciences and Primary Care, University Medical Center Utrecht, Utrecht University, Utrecht, the Netherlands; 8 Department of Orthopedic Surgery, University Medical Center Utrecht, University of Utrecht, Utrecht, the Netherlands

**Keywords:** Spinal metastases, SBRT, Surgery, Same-day treatment, RCT, TwiCs, Cost-effectiveness

## Abstract

**Background:**

Spinal metastases can lead to unremitting pain and neurological deficits, which substantially impair daily functioning and quality of life. Patients with unstable spinal metastases receive surgical stabilization followed by palliative radiotherapy as soon as wound healing allows. The time between surgery and radiotherapy delays improvement of mobility, radiotherapy-induced pain relief, local tumor control, and restart of systemic oncological therapy. Stereotactic body radiotherapy (SBRT) enables delivery of preoperative high-dose radiotherapy while dose-sparing the surgical field, allowing stabilizing surgery within only hours. Patients may experience earlier recovery of mobility, regression of pain, and return to systemic oncological therapy. The BLEND RCT evaluates the effectiveness of SBRT followed by surgery within 24 h for the treatment of symptomatic, unstable spinal metastases.

**Methods:**

This phase III randomized controlled trial is embedded within the PRospective Evaluation of interventional StudiEs on boNe meTastases (PRESENT) cohort. Patients with symptomatic, unstable spinal metastases requiring stabilizing surgery and radiotherapy will be randomized (1:1). The intervention group (*n* = 50) will be offered same-day SBRT and surgery, which they can accept or refuse. According to the Trial within Cohorts (TwiCs) design, the control group (*n* = 50) will not be informed and receive standard treatment (surgery followed by conventional radiotherapy after 1–2 weeks when wound healing allows). Baseline characteristics and outcome measures will be captured within PRESENT. The primary outcome is physical functioning (EORTC-QLQ-C15-PAL) 4 weeks after start of treatment. Secondary endpoints include pain response, time until return to systemic oncological therapy, quality of life, local tumor control, and adverse events up to 3 months post-treatment.

**Discussion:**

The BLEND RCT evaluates the effect of same-day SBRT and stabilizing surgery for the treatment of symptomatic, unstable spinal metastases compared with standard of care. We expect better functional outcomes, faster pain relief, and continuation of systemic oncological therapy. The TwiCs design enables efficient recruitment within an ongoing cohort, as well as prevention of disappointment bias and drop-out as control patients will not be informed about the trial.

**Trial registration:**

ClinicalTrials.gov NCT05575323. Registered on October 11, 2022.

## Administrative information

Note: the numbers in curly brackets in this protocol refer to SPIRIT checklist item numbers. The order of the items has been modified to group similar items (see http://www.equator-network.org/reporting-guidelines/spirit-2013-statement-defining-standard-protocol-items-for-clinical-trials/).

**Table:** 

Title {1}	Stereotactic Body radiotherapy and pedicLE screw fixatioN During one hospital visit for patients with symptomatic unstable spinal metastases: A randomized trial (BLEND RCT) using the Trials within Cohorts (TwiCs) design
Trial registration {2a and 2b}	ClinicalTrials.gov Identifier NCT05575323. Registered prospectively on October 11, 2022
Protocol version {3}	09–09-2022, version 02
Funding {4}	ZonMw number 08440012010003
Author details {5a}	Drs. E.H. Huele, Division of Imaging and Oncology, University Medical Center Utrecht, Utrecht, the Netherlands Dr. J.M. van der Velden, Department of Radiation Oncology, University Medical Center Utrecht, Utrecht, the Netherlands Drs. N. Kasperts, Department of Radiation Oncology, University Medical Center Utrecht, Utrecht, the Netherlands Drs. W.S.C. Eppinga, Department of Radiation Oncology, University Medical Center Utrecht, Utrecht, the Netherlands Dr. J.P.C. Grutters, Department for Health Evidence, Radboud University Medical Center, Nijmegen, The Netherlands Dr. B.B.M. Suelmann, Department of Medical Oncology, University Medical Center Utrecht, Utrecht, the Netherlands Drs. A.A. Weening, Department of Orthopedic Surgery, Diakonessenhuis, Utrecht, The Netherlands Dr. D. Delawi, Department of Orthopedic Surgery, St. Antoniusziekenhuis, Nieuwegein, The Netherlands Prof. S.C.C.M. Teunissen, Julius Center for Health Sciences and Primary Care, University of Utrecht, Utrecht, the Netherlands Prof. H.M. Verkooijen, Division of Imaging and Oncology, University Medical Center Utrecht, University of Utrecht, Utrecht, the Netherlands Prof. J.J. Verlaan, Department of Orthopedic Surgery, University Medical Center Utrecht, University of Utrecht, Utrecht, the Netherlands Dr. R. Gal, Division of Imaging and Oncology, University Medical Center Utrecht, University of Utrecht, Utrecht, the Netherlands
Name and contact information for the trial sponsor {5b}	UMC Utrecht
Role of sponsor {5c}	The sponsor and funder are not involved in study design; collection, management, analysis, and interpretation of data; writing of the report; and the decision to submit the report for publication.

## Introduction

### Background and rationale {6a}

With improved cancer survival rates, more patients will develop bone metastases, with the spine as the most common site [1]. Spinal metastases increase the risk for pathological fracture, spinal cord compression, and spinal instability, which can lead to devastating consequences including progressive, unremitting pain, and paralysis. The disease burden is high and significantly impairs the patients daily functioning and health-related quality of life (HRQOL) [2, 3].

Radiotherapy is the standard of care for symptomatic spinal metastases and aims to relieve pain and to obtain local control of the tumor. In addition, in case of metastatic spinal cord compression or unstable spinal metastases, surgical stabilization with or without decompression may be required [4, 5]. Currently, radiotherapy is delivered after surgery as soon as the surgical wound is healed sufficiently, which is usually after a minimum time interval of one week. This time interval delays radiotherapy-induced pain relief, improvement of mobility and return to systemic oncological therapy.

Stereotactic body radiotherapy (SBRT) enables the delivery of high-dose radiation precisely to the spinal metastasis while keeping the dose to the spinal cord and surrounding tissues, including the surgical area, low [6, 7]. Post-operative SBRT with active dose sparing of the surgical site may reduce the risk of wound complications compared to conventional radiotherapy (cRT) [8]. This makes it possible to shorten or even eliminate the time interval between surgery and radiotherapy.

However, performing SBRT post-operatively is challenging. Accurate imaging (i.e., magnetic resonance imaging (MRI) and computed tomography (CT)) is required for the precise delivery of the ablative radiation dose to a spinal metastasis. Metallic spinal implants may cause imaging artifacts, preventing accurate identification of the neural structures to be avoided during radiation, and spinal implants may limit the dose behind implants [6, 9]. Also, as MRI is time consuming, it might be uncomfortable for patients to lay on their back for a long time in the post-operative setting. Therefore, patients may benefit from preoperative SBRT. In addition to a faster treatment response, another expected advantage is an accelerated continuation or start of systemic oncological therapy, including hormonal, immune, targeted, and chemotherapy, which could lead to earlier control of (other) metastases or the primary tumor.

By providing radiotherapy followed by surgery within a single hospital stay, patients may experience faster improvement of mobility, earlier pain relief, and faster restart of systemic oncological therapy. The benefits of having both treatments in a single hospital stay are substantial considering the often short life expectancy of these patients.

### Objectives {7}

The primary objective of this study is to evaluate the effectiveness of SBRT followed by surgical stabilization with or without decompression within 24 h compared with the standard of care, which is surgical stabilization with or without decompression followed by radiotherapy (either cRT or SBRT) as soon as the wound is healed sufficiently, in terms of physical functioning at four weeks after the start of the treatment.

Secondary objectives are as follows:

1. To compare the pain response, duration of pain relief, length of hospital stay, time to return to systemic oncological therapy including hormonal, immune, targeted, and chemotherapy, neurological deterioration, adverse events (AEs) such as wound complications, HRQOL, and survival between the intervention and control group, and
2. To study the cost-effectiveness to assess whether same-day SBRT and surgery is cost-effective compared with the standard of care, from a societal perspective.

It is hypothesized that the same-day treatment procedure will result in faster recovery of mobility, less hospital visits, earlier pain relief from irradiation, and faster restart of systemic oncological therapy without an increase in wound complications due to the short interval between surgery and radiotherapy.

### Trial design {8}

**Fig. 1:**
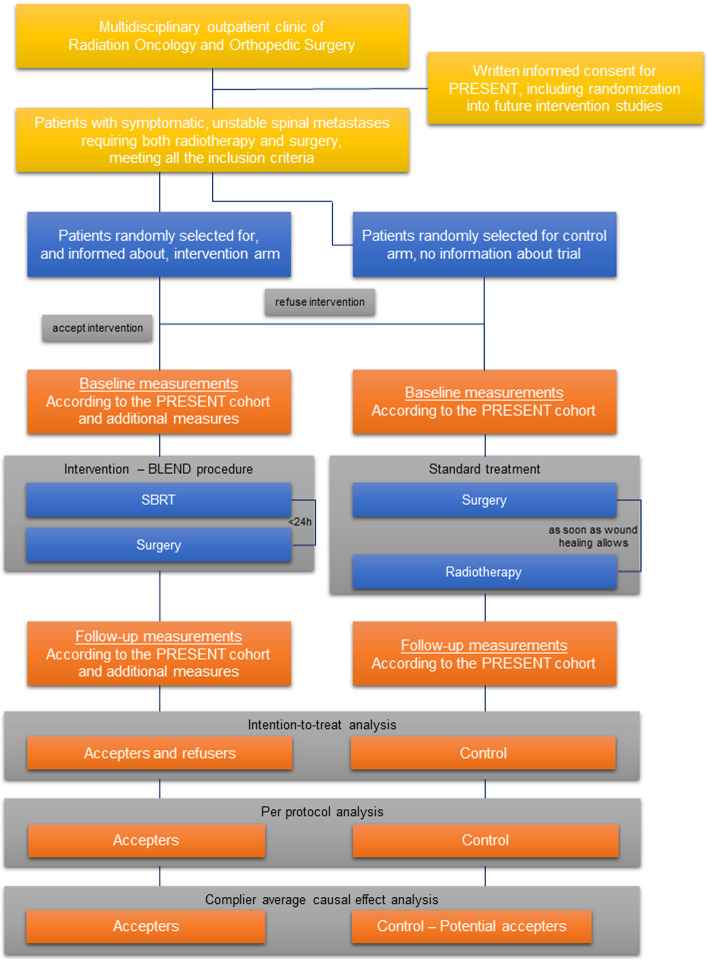
Flow chart of the study. BLEND, Stereotactic Body radiotherapy and pedicLE screw fixatioN During one hospital visit for patients with symptomatic unstable spinal metastases; PRESENT, PRospective Evaluation of interventional StudiEs on boNe meTastases; SBRT, stereotactic body radiotherapy

The BLEND RCT is a phase III randomized controlled trial within the PRospective Evaluation of interventional StudiEs on boNe meTastases (PRESENT) cohort including patients referred to the radiotherapy or orthopedic surgery department of the University Medical Center (UMC) Utrecht for the treatment of bone metastases, according to the Trials within Cohorts (TwiCs) design [1, 10]. At cohort enrolment, patients are asked consent for collection of demographic and clinical data, and patient-reported outcomes, and for randomization into future intervention studies [11]. Patients are informed that they will be randomized to either the intervention or control arm when meeting the inclusion and exclusion criteria of a future trial. When allocated to the intervention arm, they will be offered the intervention which they can accept or refuse. Informed consent is obtained from those patients accepting the intervention. When allocated to the control arm, patients will not be notified about the trial and their cohort data will be used comparatively. Patients who meet the pre-specified in- and exclusion criteria for the BLEND RCT and who have provided informed consent for randomization into future trials will be randomly allocated 1:1 to the intervention or control arm. A flow chart of the study is presented in Fig. 1.

## Methods: participants, interventions, and outcomes

### Study setting {9}

This study will be conducted within the UMC Utrecht, one of the largest academic healthcare institutions in the Netherlands, and will include patients that visit the multidisciplinary outpatient clinic of Radiation Oncology and Orthopedic Surgery.

### Eligibility criteria {10}

To be eligible, patients must meet the following inclusion criteria: (1) at least 18 years of age, (2) having symptomatic (cervical, thoracic and/or lumbar) spinal metastases from solid tumors and (impending) spinal instability requiring surgical stabilization (with or without decompression) and radiotherapy, (3) histologic proof of malignancy or radiographic/clinical characteristics indicating malignancy beyond reasonable doubt, (4) radiographic evidence of spinal metastases, (5) fit for (radio)surgery, (6) participation in PRESENT cohort, including consent for providing PROMs and randomization into future trials, and (7) written informed consent. Potential patients will be excluded when (1) SBRT cannot be delivered, (2) routine surgical stabilization with or without decompression and/or radiotherapy cannot be performed (e.g., multiple spinal metastases requiring surgical bridging of more than five vertebral levels and/or requiring radiotherapy on more than one location), (3) prior surgery or radiotherapy to the index level(s), (4) multiple myeloma, (5) neurological deficits (American Spinal Injury Association (ASIA) impairment scale grade A, B, or C) or partial neurological deficits (ASIA grade D) with rapid progression (hours to days), (6) current treatment with Bevacizumab and other medication with long half-life that interferes with radiotherapy, (7) life expectancy of less than 3 months, and/or (8) pregnancy.

### Who will take informed consent? {26a}

Patients will be informed at the outpatient innovation clinic by the research team about the PRESENT cohort. Patients are asked informed consent for the collection of demographic and clinical data and patient-reported outcome measures (PROMs) at regular time points. Patients can provide additional consent for randomization into future intervention studies (i.e., broad consent) [11]. Patients who provided broad consent and who meet the eligibility criteria will be randomly allocated 1:1 to the intervention or control arm stratified by renal cell carcinoma versus other primary tumor histologies. Patients allocated to the intervention arm will be offered the intervention, which they may accept or refuse. Upon acceptance, informed consent will be obtained by a member of the research team.

### Additional consent provisions for collection and use of participant data and biological specimens {26b}

Not applicable, no additional samples will be collected.

### Interventions

#### Explanation for the choice of comparators {6b}

According to the TwiCs design, the control group will receive standard of care. Current standard of care is surgical stabilization with or without decompression followed by radiotherapy (either cRT or SBRT) as soon as the wound is healed sufficiently, which is usually after a minimum time interval of 1 week. Inherent to the TwiCs design, control patients will not be informed about the trial and that they serve as a control. Previous research showed that most patients were positively or neutrally to having served as controls without notification of being selected for the control group [12]. By not informing controls, disappointment bias and contamination (i.e., control patients receiving the experimental intervention) may be prevented.

#### Intervention description {11a}

Patients who accept the intervention will receive same-day SBRT and surgery. Before treatment, patients will undergo an extended MRI and CT scan in treatment position for planning purposes. Next, cone beam CT (CBCT) before and during dose delivery is made to correct for position deviations. Then, patients will receive SBRT, which will be a single high dose of at least 18 Gray (Gy); this dose can be increased to 21 Gy in patients with radioresistant tumors, to the affected vertebra. Dose constraints are set for the organ at risks based on institution specific guidelines. Within 24 h after SBRT, surgical stabilization with or without decompression will be performed according to the standard of care and on an elective base. The type of (pedicle screw) fixation will be decided by the surgeon depending on the need for surgical decompression, vertebral levels to be included in the construct, and the bone quality.

The control arm and patients who refused to undergo the intervention will receive the standard of care. Currently, the standard of care for patients with unstable spinal metastases is surgical stabilization with or without decompression followed by cRT or SBRT as soon as the surgical wound has healed sufficiently. Before surgery, all patients will undergo an extended planning MRI in radiotherapy treatment position. After surgery and before radiotherapy, when the wound is healed sufficiently, a planning CT is made and another MRI to see if the neurological structures have moved after surgery. Pre- and postoperative MRI are mutually registered to yield information on all relevant structures for planning assessing dose distribution using volumetric modulated arc therapy (VMAT). Also, CBCT data will be acquired to correct for translations or rotations, and if correct in subsequent CBCTs, radiotherapy will be delivered.

#### Criteria for discontinuing or modifying allocated interventions {11b}

If it turns out that patients do not meet the inclusion criteria after randomization (e.g., proper planning of the radiation dose is not possible without meeting the constraints to the organs at risk, especially the spinal cord), patients will be excluded from the BLEND RCT and they will receive standard of care. The intervention or standard of care can be discontinued or modified for urgent medical reasons. Patients can withdraw consent for the intervention and/or participation in the PRESENT cohort (and hence the BLEND RCT) at any time for any reason if they wish to do so without any consequences.

#### Strategies to improve adherence to interventions {11c}

As the intervention includes one clinical treatment procedure, no specific strategies exist to improve intervention adherence. However, routine outcome measures from the PRESENT cohort will be used for effect estimation. To minimize missing outcomes on the primary endpoint, a member of the research team will call patients who do not return a questionnaire within 1 week to request the patient to complete the questionnaire and to ask out their response on the primary outcome.

#### Relevant concomitant care permitted or prohibited during the trial {11d}

No concomitant care, interventions, and medications will be prohibited during the trial.

#### Provisions for post-trial care {30}

There are no provisions for post-trial care because we do not expect study related (serious) AEs beyond the risk of unrelated (S)AEs due to the natural course of the disease and (S)AEs related to standard of care. A previous first-in-man study including 13 patients demonstrated that the delivery of SBRT followed by surgical stabilization with or without decompression within 24 h is safe and feasible, and no wound complications were observed within 90 days following treatment [13].

#### Outcomes {12}

The primary outcome is difference in physical functioning four weeks after the start of the treatment (i.e., surgery or radiotherapy) between the intervention and control arm, which will be measured with the European Organization for Research and Treatment of Cancer (EORTC) Quality of Life Questionnaire (QLQ) Core 15 Palliative Care (C15-PAL) [14].

Secondary outcomes include the following:

- Pain response at 2, 4, 6, and 8 weeks and 3 months
- Duration of pain relief as measured by the Brief Pain Inventory (BPI) [15, 16]
- Change in HRQOL as measured by the EORTC QLQ-C15-PAL and EORTC QLQ Bone Metastases 22 (BM22) from baseline to 4 and 8 weeks and 3 and 6 months [14, 17, 18]. For the present study, we will focus on overall QOL and the two functional scales (physical and emotional functioning) of the EORTC QLQ-C15-PAL, and the functional interference and psychosocial aspects domains of the EORTC QLQ-BM22 [17, 18]
- Duration of the hospital stay in days
- Days until return to systemic oncological therapy, including hormonal, immune, targeted, and chemotherapy
- Occurrence of neurological deterioration, defined as deterioration of more than one grade on the American Spinal Cord Injury Association (ASIA) scale as measured by physical examination by a neurologists or trained ASIA physician [19, 20]
- Local control according to routine imaging and the electronic patient chart
- AEs, defined as any undesirable experience occurring to a patient during the study that may be related to the experimental treatment procedure
- Progression-free survival and overall survival
- Resource use, such as doctor visits, medication, hospital admissions, and surgical interventions, as well as out-of-pocket expenses such as for over-the-counter drugs and travel costs, will be recorded using the iMTA Medical Consumption Questionnaire (iMCQ) at 3 and 6 months [21]
- Cost-effectiveness

### Participant timeline {13}

**Table:** 

	Before first consultation with radiation oncologist and orthopedic surgeon	Short after first consultation with radiation oncologist and orthopedic surgeon	Regular PRESENT measurements						
			Baseline	2 weeks	4 weeks	6 weeks	8 weeks	3 months	6 months +
**Enrolment**									
Informed consent PRESENT cohort	X								
Eligibility screening BLEND RCT	X								
Randomization BLEND RCT	X								
Informed consent BLEND RCT (upon acceptance of intervention)		X							
**Interventions**									
BLEND procedure		X (same-day RT and surgery)							
Standard of care		X (surgery followed by RT when the wound healed sufficiently, usually > 1 week)							
**Assessment**									
PROMs used for BLEND RCT effect estimation primary outcome					X				
PROMs used for BLEND RCT effect estimation secondary outcome			X	X	X	X	X	X	X

#### Sample size {14}

Four weeks after the start of treatment, patients in the control group may just have received radiotherapy or still need to undergo radiotherapy. Therefore, we assume that they do not experience a pain response yet at that time point. From the PRESENT cohort, we know that patients who did not experience a pain response after radiotherapy had a physical functioning mean score of 43.6, 4 weeks after the start of radiotherapy.

A recent study reported a minimal clinically important difference (MCID) of 13 points on the EORTC QLQ-C15-PAL physical functioning scale in patients with bone metastases undergoing radiotherapy [22]. To detect a 13 point difference with an 80% power, a two-sided *α* of 5% and standard deviation of 22, 45 patients are needed in each arm. We expect that 90% of the patients offered the intervention will accept the offer. To compensate for refusal, questionnaire nonresponse, and possible dropouts, we will increase the sample size by 10%, resulting in a total sample size of 100 patients.

### Recruitment {15}

For this study, no recruitment strategies will be used as eligible patients will be identified from the PRESENT cohort.

### Assignment of interventions: allocation

#### Sequence generation {16a}

A computer-generated allocation sequence will be created using random permuted blocks of patients. Randomization will be stratified by spinal metastases from renal cell carcinoma versus other histologies.

#### Concealment mechanism {16b}

Patients will be randomized using Castor (Amsterdam, The Netherlands, www.castoredc.com), an electronic data capture system. Allocation concealment will be ensured as Castor will not release the randomization code until the patient has been recruited into the trial.

#### Implementation {16c}

The research team will screen patients on eligibility. If a patient appears eligible, the research team will perform online randomized allocation.

### Assignment of interventions: blinding

#### Who will be blinded {17a}

Patients randomized to the control group will not be informed about the trial and receive standard of care. Hence, patients in the control group will be blinded to group allocation. They will even be unaware of the existence of this trial. In contrast, patients randomized to the intervention group will be informed about the intervention which they can accept or refuse. It is not possible to blind researchers and clinicians for group allocation.

#### Procedure for unblinding if needed {17b}

Not applicable. According to the TwiCs design, patients in the control group will not be informed about their role as a control in this trial.

### Data collection and management

#### Plans for assessment and collection of outcomes {18a}

According to the TwiCs design, demographic and clinical data and regular PROMs collected in the PRESENT cohort will be used for effect estimation. In general, patients fill out the EORTC QLQ-C15-PAL, the EORTC QLQ-BM22, the EuroQol (EQ) 5D-5L, and the BPI at baseline (upon cohort enrolment, i.e., before the start of treatment), 4 and 8 weeks, 3 and 6 months after initial treatment, and every 6 months thereafter. At 2 and 6 weeks, patients fill out an additional BPI [1, 14–18, 23]. Patients can choose to fill out the questionnaires on paper or online.

The primary outcome is physical functioning at 4 weeks that will be assessed using the EORTC QLQ-C15-PAL, a 15-item HRQOL questionnaire representing overall QOL, two functional scales (physical and emotional functioning), and six symptom scales (nausea, loss of appetite, dyspnea, constipation, sleeping difficulties, and fatigue) [14]. Secondary, change in HRQOL from baseline to 4 and 8 weeks and 3 and 6 months will be measured using the EORTC QLQ-C15 (overall QOL, physical and emotional functioning) and EORTC QLQ-BM22 (functional interference and psychosocial aspects). The EORTC QLQ-BM22 is a 22-item questionnaire representing four domains: painful sites, pain characteristic, functional interference, and psychosocial aspects [17, 18]. Items of the EORTC QLQ-C15 and -BM22 will be rated on a Likert scale ranging from 1 (not at all) to 4 (very much) with the exception of the overall QOL item that will be rated on a 7-point Likert scale ranging from 1 (very poor) to 7 (excellent). Scale scores will be linearly transformed to a 0–100 scale where a higher score on the overall QOL and functioning scales indicates better functioning, and on the symptom scales an increase of symptoms. The EORTC QLQ-C15 and -BM22 has been validated in patients with painful bone metastases or advanced cancer [17, 18, 24].

The duration of pain relief up to three months will be measured by the BPI which is a multidimensional tool to evaluate cancer pain [15, 16]. The BPI consists of eleven items representing two domains: pain perception and interference by pain. Items can be rated using a scale from 0 (no pain/interference) to 10 (maximum pain/interference). The BPI has been validated in patients with painful bone metastases [16]. Duration of pain relief will be defined as the time between response and progression or end of follow-up.

Pain response at 2, 4, 6, and 8 weeks and 3 months is defined as [1] a decline in worst pain score of at least two points on an NRS of ten at the treated site without increase in analgesic use or [2] an analgesic decrease of at least 25% without an increase in pain score [25–27]. Worst pain score will be measured with the BPI [15, 16].

Information on duration of the hospital stay in days, days until return to systemic oncological therapy, local control, and progression free survival and overall survival will be captured from the electronic health record. Also, occurrence of neurological deterioration, defined as deterioration of more than one grade on the American Spinal Cord Injury Association (ASIA) scale as measured by physical examination by a neurologists or trained ASIA physician, will be captured from the electronic health record [19, 20].

AEs are defined as any undesirable experience occurring to a patient during the study that may be related to the experimental treatment procedure. AEs during hospital stay will be evaluated using the most recent version of the National Cancer Institute Common Toxicity Criteria of Adverse Events (CTCAE; version 5.0, version 6.0 when available at the start of the study). AEs after hospital discharge reported spontaneously by the patient or observed by the research team will be recorded up to 3 months after treatment.

For cost-effectiveness evaluation, resource use, costs, and quality-adjusted life years (QALYs) are measured for each patient in both randomization groups over the follow-up period. Resource use such as doctor visits, medication, hospital admissions, and surgical interventions, as well as out-of-pocket expenses such as for over-the-counter drugs and travel costs, will be captured using the iMCQ at 3 and 6 months [21]. Where relevant, questionnaire responses will be verified or completed by data from the medical records. Total costs for each patient will be calculated by multiplying the resource use with unit costs. Unit costs will be calculated according to the guidelines for costing research. QALYs will be calculated from the EQ-5D-5L, using the area under the curve approach [23].

#### Plans to promote participant retention and complete follow-up {18b}

Patients will be contacted by phone when they do not complete the questionnaire at four weeks follow-up that captures the primary outcome within three days. The researcher will ask the patient to verbally rate the items that are necessary to evaluate the primary endpoint. In addition, the researcher will remind the patient to complete this questionnaire. Patients who fill out the questionnaires online will receive a reminder if they do not return the questionnaire within 1 week. Patients who fill out the questionnaires on paper will be called by the research team to keep them engaged. Other secondary outcomes are based on information from the medical health record and, therefore, this data will be near to complete.

Patients who withdraw from this trial will be followed up in the PRESENT cohort if they do not withdraw from the PRESENT cohort. PRESENT data can be used for outcome evaluation. If they withdraw from the PRESENT cohort, their data up to withdrawal will be retained and utilized in analysis.

#### Data management {19}

Demographic and clinical data will be collected in the electronic data capture system Castor (Amsterdam, The Netherlands, www.castoredc.com). The database includes multiple build-in skips and data validation checks to promote data quality, and an electronic audit trial log of all study event is stored. The data will be managed by a data manager and the research team will have access to the data. Access will be managed by authorization of accounts. PROMs will be sent out to the patients using PROFILES, which stands for Patient Reported Outcomes Following Initial treatment and Long term Evaluation of Survivorship (www.profielstudie.nl). Patients can complete the questionnaire on paper or online. Hardcopy questionnaires will be entered into PROFILES and physically stored in a secure archive of the study site. For other study files, a local secured research folder on the network drives of the study site will be used to be sure that only authorized personnel have access to the data.

#### Confidentiality {27}

The data will be handled confidentially and in compliance with the Dutch Personal Data Protection Act (De Wet Bescherming Persoonsgegevens). Research data will be coded by an identification number that will not be based on identifiable personal data. The subject identification code list to personal data will be only accessible to authorized personnel. Informed consent forms will be stored in a secure archive of the study site.

#### Plans for collection, laboratory evaluation, and storage of biological specimens for genetic or molecular analysis in this trial/future use {33}

Not applicable. No biological specimens will be collected.

### Statistical methods

#### Statistical methods for primary and secondary outcomes {20a}

The primary outcome is physical functioning 4 weeks after the start of the treatment, which will be expressed as a score on a 0–100 scale. First, we will calculate within-group changes from baseline to 4 weeks after the start of the treatment, which will be expressed as mean changes with corresponding 95% confidence intervals. Linear regression analysis or analysis of covariance (ANCOVA) following the intention-to-treat principle will be performed to compare the intervention arm with the control arm in terms of physical functioning, controlling for baseline physical functioning, age, Karnofsky performance score, and medication use. Differences between treatments and 95% confidence intervals will be calculated.

The proportion of patients in the intervention and control arm reporting a pain response will be compared by Fisher’s exact test or *χ*^2^ test.

Within-group changes in HRQOL from baseline to 4 and 8 weeks and 3 and 6 months will be expressed as mean change with corresponding 95% confidence intervals. We will assess between-group differences in HRQOL as a continuous outcome using linear regression analysis. The proportion of patients with a clinically relevant improvement or deterioration, i.e., an increase or decrease, respectively, of at least 10 points will be compared between the intervention and control arm at every time point by Fisher’s exact test or *χ*^2^ test [24, 28]. In addition, the proportion of patients reporting a clinically relevant improvement at any time point within 3 months will be compared.

A cost-effectiveness analysis will be performed to assess whether same-day SBRT and surgery is cost-effective compared with the standard of care, from a societal perspective. If same-day SBRT and surgery is more costly and more effective than the standard of care, an incremental cost-effectiveness ratio (ICER) will be calculated by dividing the extra QALYs by the extra effects. This will give an estimation of the extra costs that are needed to gain one QALY. If this is below the cost-effectiveness threshold, same-day SBRT and surgery is deemed cost-effective. If either same-day SBRT and surgery or the standard of care is less costly and more effective than the standard of care, no ICER is necessary to determine cost-effectiveness. Uncertainty surrounding the costs, effects and ICER will be addressed by means of bootstrapping. Overall mean and median costs will be compared across the randomization groups and, where relevant, differences will be calculated inclusive of 95% confidence intervals. Also, a budget impact analysis (BIA) will be performed using the ZonMw tool to address the question whether same-day SBRT and surgery contributes to an affordable and more efficient and sustainable healthcare system. Data that will be used reflect the size and characteristics of the population, the current and new treatment mix, the efficacy of same-day SBRT and surgery and the standard of care, the resource use, and costs for the treatments and symptoms (https://www.zonmw.nl/nl/onderzoek-resultaten/doelmatigheidsonderzoek/budget-impact-analyse-bia/).

#### Interim analyses {21b}

An interim analysis will be performed when 50% of the patients have been randomized and completed the 4 weeks follow-up period. This analysis will be performed to assess how many patients complete the primary outcome measurement and to estimate the drop-out rate. If the drop-out rate is higher than expected (i.e., > 10%), we should consider to update the sample size to prevent an underpowered study [29].

#### Methods for additional analyses (e.g., subgroup analyses) {20b}

Not applicable. There will be no additional analyses (e.g., subgroup and adjusted analyses).

#### Methods in analysis to handle protocol non-adherence and any statistical methods to handle missing data {20c}

Analysis will be based on the intention-to-treat principle. However, in accordance with the TwiCs design, patients who are selected for the intervention arm may refuse the intervention. In case of a high refusal rate (i.e., > 15%), we will also conduct a per protocol (PP) analysis including patients who completed the treatment as allocated. However, refusal may be selective, e.g., patients who are less physically fit may refuse the intervention. As a result, PP analysis may under- or overestimate the treatment effect. We will also perform a complier average causal effect (CACE) analysis to estimate the treatment effect among “compliers,” i.e., patients that accepted and received the intervention offered compared with hypothetical control patients who, if offered the intervention, would have accepted.

In the palliative oncology setting, questionnaire non-response is more likely due to inability to complete questionnaires as a result of for example fatigue and high distress or filling in questionnaires may be too burdensome. The proportion and patterns of missing data for the primary and secondary outcomes will be investigated. If needed, multiple imputation methods will be used to handle missing data under the assumption that data is missing at random. If the primary outcome data is determined to be missing not at random, a best–worst and worst-best case sensitivity analyses will be performed.

#### Plans to give access to the full protocol, participant-level data, and statistical code {31c}

Following publication of the study results, datasets containing de-identified patient-level data will be made accessible upon reasonable request to the principle investigator or the corresponding author. Data will be accessible in a digital workspace on a cloud-based platform.

### Oversight and monitoring

#### Composition of the coordinating center and trial steering committee {5d}

The principle investigator, coordinating investigators, spine surgeon, and radiation oncologist are responsible for the monitoring of study process. The project group will meet every 2 weeks to discuss the progress of the trial. Ad hoc meetings will be scheduled to address time-sensitive issues as they arise.

#### Composition of the data monitoring committee, its role and reporting structure {21a}

Data monitoring will be performed by an independent monitor who will monitor the progression of the study, check the presence of informed consent, review the presence of standard operating procedures (SOPs) and adherence to the study protocol, SOPs and the Medical Research Involving Human Subjects Act (WMO), and verify accuracy and completeness of the data. The monitor will brief any issues to the study team, who will address and resolve these issues and report back to the monitor.

#### Adverse event reporting and harms {22}

Adverse events during hospital stay will be evaluated using the most recent version of the CTCAE (version 5.0, version 6.0 when available at the start of the study). Due to the natural course of the disease, we expect that many patients with spinal metastases suffer from study unrelated AEs. Therefore, only study-related AEs with grade 2 or higher (either expected or unexpected) reported spontaneously by the patient or observed by the research team will be recorded up to three months after treatment.

Grade 3 or higher adverse events are considered serious (S) AEs. (S)AEs that are common in this patient category include urinary tract infections, delirium, pressure sores, and opioid-related adverse drug events and will be recorded but not reported to the ethics committee. Study-related SAEs within 3 months after start of treatment that are life threatening or that resulted in death will be directly reported (within 7 days) to the ethics committee. Other study-related SAEs will be reported to the ethics committee within 15 days. SAEs unrelated to the study will not be directly reported to the ethics committee but documented in the yearly progress report.

#### Frequency and plans for auditing trial conduct {23}

There will be on-site monitor visits before the start of the study, during the conduct of the study and at the end of the trial.

#### Plans for communicating important protocol amendments to relevant parties (e.g., trial participants, ethical committees) {25}

Substantial amendments are changes to the study that may have a significant effect on the safety or rights of the patient, the robustness of the generated data, and/or the conduct of the study. Substantial amendments will be submitted to the ethics committee for approval. After approval, changes will be communicated to the study team and the trial registry will be updated.

#### Dissemination plans {31a}

The trial results will be disseminated through publication in open access peer-reviewed scientific journals and presentation at key scientific (inter)national conferences. Results will also be available on the project page of the website of the funder.

## Discussion

This trial investigates same-day SBRT and surgery in patients with symptomatic spinal metastases to achieve faster improvement of mobility, earlier pain relief, and faster continuation of systemic oncological therapy, compared to the standard of care, which is surgery followed by radiotherapy (either cRT or SBRT). Same-day treatment may significantly decrease the burden for the patients which may be substantial considering the often limited life expectancy of these patients. In addition, same-day treatment may overcome limitations related to the standard of care, such as an increased risk of wound complications when radiotherapy is delivered too early. Furthermore, metallic spinal implants may cause imaging artifacts and may limit the dose behind the implants [6, 9, 30]. Also, as MRI is time consuming, it might be uncomfortable for patients to lay on their back for a long time in the post-operative setting.

A systematic review reported mixed findings on the association between preoperative radiotherapy and wound complications [31]. The authors recommended a time interval of at least 7 days between radiotherapy and surgery but that this interval can be reduced with SBRT. Nevertheless, a previous first-in-man study including thirteen patients demonstrated that the delivery of SBRT followed by surgical stabilization with or without decompression within 24 h is safe and feasible, and no wound complications were observed [13]. There is no literature available on the effect of same-day SBRT and surgery on patient-reported outcomes including physical functioning, pain, and HRQOL.

We will use the TwiCs design as it has several advantages over the conventional RCT design. One of the shortcomings of the conventional RCT design when investigating this research question is that blinding is not possible and patients know whether they receive the intervention or not [32]. Assumably, patients prefer the new and promising intervention over the control arm, and they may be disappointed when allocated to the control group. As a consequence, patients do not want to participate because of the concept of randomization or (a selective group of) patients may drop-out after allocation to the control group. In addition to accrual problems that are common in the palliative setting, it may be difficult to reach the target sample size. Furthermore, patients that want to participate in an RCT may be a selective group of patients that differs from the target population, which might hamper generalizability. This may be prevented with the TwiCs design since we will recruit patient from the PRESENT cohort.

In the UMC Utrecht, three trials in the oncological setting using the TwiCs design were successfully completed [33–38]. By using this design, we experienced easier patient recruitment and improved representativeness of the study sample and generalizability of results [37, 39, 40]. In addition, patients allocated to control will not be notified about the trial and, hence, patients will not receive information about a promising intervention that they will not receive preventing disappointment bias. Moreover, in a conventional RCT, patients are informed about an innovative treatment which could induce hope for better results. When allocated to the control arm and knowing that they do not receive the promising new intervention, patients could rate their outcomes more negatively. Therefore, the TwiCs design could be especially relevant in trials with subjective outcomes such as physical functioning and HRQOL. Also, the design provides unique insight into the applicability and acceptability of same-day SBRT and surgery [36].

We expect that this trial will eventually result in change of current clinical practices and evidence-based treatment recommendations for spine oncology.

## Trial status

The study protocol was approved by the ethics committee NedMec, the Netherlands, on 19 September 2022 (protocol version 02). Recruitment began November 2022 and is expected to end June 2026.

## Data Availability

Following publication of the study results, datasets containing de-identified patient-level data will be made accessible upon reasonable request to the principle investigator or the corresponding author. Data will be accessible in a digital workspace on a cloud-based platform.

## Abbreviations

AE: Adverse event
ANCOVA: Analysis of covariance
ASIA: American Spinal Injury Association
BIA: Budget impact analysis
BLEND: Stereotactic Body radiotherapy and pedicLE screw fixatioN During one hospital visit for patients with symptomatic unstable spinal metastases
BM: Bone metastases
BPI: Brief Pain Inventory
CACE: Complier average causal effect
CBCT: Cone beam computed tomography
cRT: Conventional radiotherapy
CT: Computed tomography
CTCAE: Common Toxicity Criteria of Adverse Events
EORTC: European Organization for Research and Treatment of Cancer
EQ: EuroQol
Gy: Gray
HRQOL: Health-related quality of life
ICER: Incremental cost-effectiveness ratio
iMCQ: IMTA Medical Consumption Questionnaire
MCID: Minimal clinically important difference
MRI: Magnetic resonance imaging
PAL: Palliative care
PP: Per protocol
PRESENT: PRospective Evaluation of interventional StudiEs on boNe meTastases
PROFILES: Patient Reported Outcomes Following Initial treatment and Long term Evaluation of Survivorship
PROMs: Patient-reported outcome measures
QALYs: Quality-adjusted life years
QLQ: Quality of Life Questionnaire
RCT: Randomized controlled trial
RT: Radiotherapy
SAE: Serious adverse event
SBRT: Stereotactic body radiotherapy
SOP: Standard operating procedure
TwiCs: Trials within Cohorts
UMC: University Medical Center
VMAT: Volumetric modulated arc therapy
WMO: Medical Research Involving Human Subjects Act
